# CCM111, the water extract of *Antrodia cinnamomea*, regulates immune-related activity through STAT3 and NF-κB pathways

**DOI:** 10.1038/s41598-017-05072-y

**Published:** 2017-07-07

**Authors:** In-Yu Lin, Min-Hsiung Pan, Ching-Shu Lai, Ting-Ting Lin, Chiung-Tong Chen, Tao-Sheng Chung, Chien-Lung Chen, Chen-Huan Lin, Wu-Chang Chuang, Ming-Chung Lee, Ching-Che Lin, Nianhan Ma

**Affiliations:** 1 Department of Biomedical Sciences and Engineering; Institute of Systems Biology and Bioinformatics, National Central University, Taoyuan, Taiwan; 2 Institute of Food Science and Technology, National Taiwan University, Taipei, Taiwan; 3 Department of Seafood Science, National Kaohsiung Marine University, Kaohsiung, Taiwan; 4 Institute of Biotechnology and Pharmaceutical Research, National Health Research Institutes, Zhunan, Miaoli Taiwan; 5 Division of Radiation Oncology, Landseed Hospital Taoyuan, Taoyuan, Taiwan; 6 Division of Nephrology, Landseed Hospital Taoyuan, Taoyuan, Taiwan; 7 Brion Research Institute of Taiwan, New Taipei City, Taiwan; 8 Department of Medical Research, China Medical University Hospital, China Medical University, Taichung, Taiwan; 9 Department of Health and Nutrition Biotechnology, Asia University, Taichung, Taiwan

## Abstract

*Antrodia cinnamomea* (AC) exhibits many bioactivities, including anti-inflammatory, anti-cancer, and hepatoprotection activities. Many researchers have studied the functions of the components or fractions of AC, but the functions of the original extractions of AC have not been studied. In addition, the detailed relationship between AC and immune-related signaling pathways is unclear. In this study, we screened the effects of CCM111, which is the extract of AC, on seven immune-related signaling pathways and further investigated whether CCM111 can influence inflammation. Interestingly, our results showed that CCM111 significantly inhibited the IL-6-stimulated STAT3 pathway and the LPS-stimulated NF-κB pathway in macrophages. CCM111 also decreased the phosphorylation of STAT3, Tyk2 and the nuclear translocation of p65. Moreover, CCM111 and F4, a fraction of CCM111, down-regulated nitric oxide (NO) production, the protein levels of iNOS and COX-2, and inflammatory cytokines in macrophage cells. Therefore, our study suggested that CCM111 has the potential to be developed as an effective anti-inflammatory agent.

## Introduction

Inflammation is an innate immune response and affects many human diseases, including cancers. Some studies have reported that anti-inflammatory activity decreases the risk of human diseases^1, 2^. Inflammation involves a variety of immune cells. Macrophages are one of the types of immune cells critical in inflammation that can be induced by pathogen-associated molecular patterns (PAMPs) such as lipopolysaccharide (LPS)^3^, a major component of gram-negative bacteria membranes, to secrete many pro-inflammatory cytokines including TNF-α and IL-6. Moreover, inducible nitric oxide synthase (iNOS) and cyclooxygenase-2 (COX-2) are two important enzymes involved in the inflammatory response^4^. iNOS can generate nitric oxide (NO), and excessive NO is linked to inflammation and septic shock^5^. COX-2 is the major enzyme that generates prostaglandin (PGE_2_), which is significantly increased in inflamed tissue and sustains the inflammation responses^6^.

Inflammation responses are regulated by many signal transduction pathways, such as the nuclear factor-kappa B (NF-*κ*B), the signal transducer and activator of transcription protein (STAT), and the toll-like receptor (TLR) pathways. NF-*κ*B regulates the expression of many pro-inflammatory cytokines and chemokines, and it regulates cell proliferation and survival^7^. NF-*κ*B can be activated by cytokines, including TNF-α. IL-1β and LPS regulate the promoter regions of iNOS and COX-2^8, 9^. STATs are transcription factors that regulate immune response, cell differentiation, cell growth and cell survival. STAT pathways activation are regulated by Janus kinases, such as JAK1, JAK2, JAK3 and TYK2, which are intracellular kinases^10^. In response to IFN-γ stimulation, the phosphorylation of the intracellular domain of the IFNGR receptor serves as a STAT1 docking site. STAT1 forms homodimers or heterodimers with STAT3 that then bind to the gamma activated site (GAS) promoter, which is necessary for expression of iNOS in IFNγ- and LPS-induced inflammation^11, 12^. STAT2 can activate both IFN-α/β and IFN-λ to protect against viral infections^13^. Type I interferons activate the formation of the STAT1/2 heterodimer, which binds to IFN-stimulated response elements (ISREs) and initiates antiviral responses^14, 15^. STAT3 activates many inflammation-relative cytokines such as IL-5, IL-6 and COX-2^16, 17^. STAT3 regulates innate immune responses such as granulopoiesis, the proliferation and migration of neutrophils^17^. In addition, persistent activation of the STAT3 pathway has been reported to connect with cancer-mediated inflammation^18^. TLRs are a family of sensors for the innate immune system, and they induce host immune responses through recognizing PAMPs^19, 20^. TLR2 specifically recognizes peptidoglycan (PGN), which is a cell wall component of gram-positive bacteria. The activation of TLR2 induces pro-inflammation cytokines and type I interferons^21, 22^. TLR3 specifically recognizes polyinosinic–polycytidylic acid (poly(I:C)), which is a viral double-stranded RNA analog. The activation of TLR3 up-regulates type 1 interferons and induces dendritic cell maturation^23^. TLR4 specifically recognizes lipopolysaccharide (LPS), which is a component of gram-negative bacteria. The activation of TLR4 induces pro-inflammation cytokines such as TNF-α production in macrophage cell lines^24^. Stimulation through TLR2, TLR3, and TLR4 receptors by PAMPs activates the NF-κB and STAT3 signaling pathways and further induces the production of many pro-inflammatory cytokines^25–28^.

*Antrodia cinnamomea* (AC; synonym: *Antrodia camphorata*) is a unique herb found in Taiwan, and it is used as a traditional medicine for immune modulation or liver protection^29, 30^. Previous studies have reported that the whole ethanol extract of AC contains anti-inflammatory functions^31–33^. The extraction components of AC inhibited LPS-induced expression or production of cytokines, iNOS, COX-2, IL-6, and NO by reducing NF-κB signaling in murine macrophage cells^34–37^. The components of AC also alleviated the carrageenan-induced inflammation response, NO, and TNF-α production and ameliorated imiquimod-induced skin inflammation *in vivo* ^38, 39^. However, the molecular mechanisms of the anti-inflammatory activity of the crude water extract of AC, which is obtained by the clinical traditional method, remain unclear. In this study, we prepared a crude water extract of AC, referred to as CCM111, and investigated its role in the modulation of immune responses by dissecting its mechanisms in signal transduction pathways and cytokines.

Our results demonstrated that CCM111 significantly inhibits the activity of the STAT3, NF-κB and TLR4 signaling pathways and that it moderately inhibits the STAT1/2 pathway. We also found that CCM111 decreases nitric oxide (NO) production and the protein levels of iNOS and COX2, and we found that it also decreases many inflammation-related cytokines in mouse macrophage cells. Furthermore, a subfraction of CCM111, F4, decreases NO production, iNOS, COX-2, and the phosphorylation of Tyk2. Therefore, our study suggested that the extracts of AC obtained by traditional extraction methods have anti-inflammatory functions and that CCM111 has the potential to be an anti-inflammatory agent.

## Results

### Toxicity of CCM111 and chemical fingerprint analysis of CCM111 by HPLC-UV

**Figure 1:**
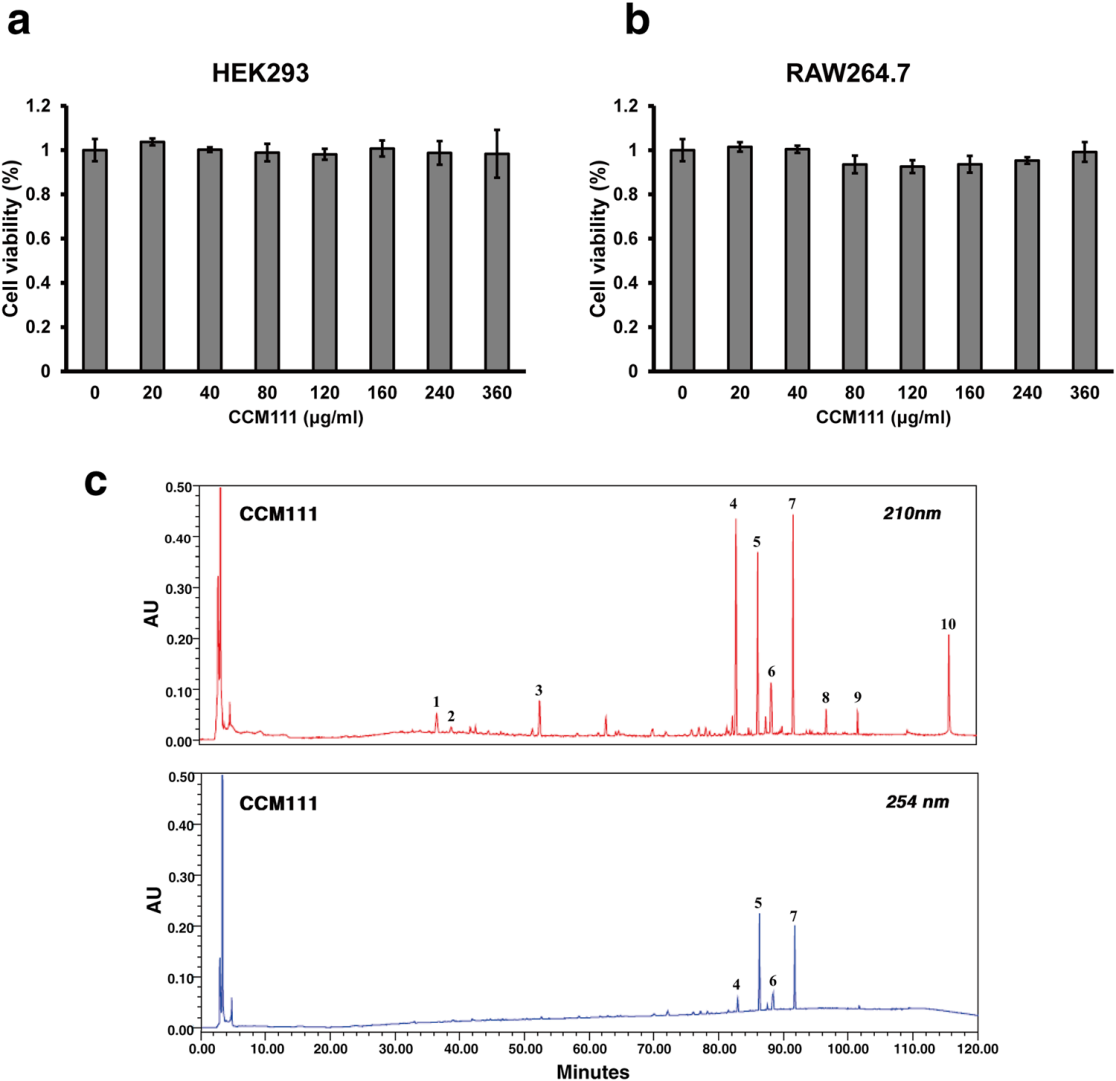
Toxicity of CCM111 and chemical fingerprint analysis of CCM111 by HPLC-UV. (**a**) and (**b**) HEK293 or RAW264.7 cells were treated separately with different concentrations of CCM111 (0, 20, 40, 80, 120, 160, 240, and 360 μg/ml) for 24 hours. The survival of cells was detected by the Alamar blue assay. Results were obtained from three independent replicates. (**c**) The HPLC-UV method was performed to establish the quality control of CCM111. The absorption of UV wavelengths was measured at 210 nm and 254 nm.

CCM111 is the water extract of *Antrodia cinnamomea*, and we investigated its immune functions. We first examined if CCM111 induced toxicity in HEK293 human epithelial cells and RAW 264.7 macrophages. The cell numbers were not affected after CCM111 treatment for 24 hours (Fig. 1a,b). We next established procedures to standardize the quality control of CCM111 by detecting its HPLC-UV fingerprint. This map showed three major peaks and seven minor peaks at 210 nm as well as two major peaks and two minor peaks at 254 nm (Fig. 1c). The peaks were further identified by LC/MS as follows: 4, antrodin D (82.9 min); 5, antrodin C (86.3 min); and 6, antrodin A (88.3 min).

### Effects of CCM111 on immune-related pathways in HEK293 cells

**Figure 2:**
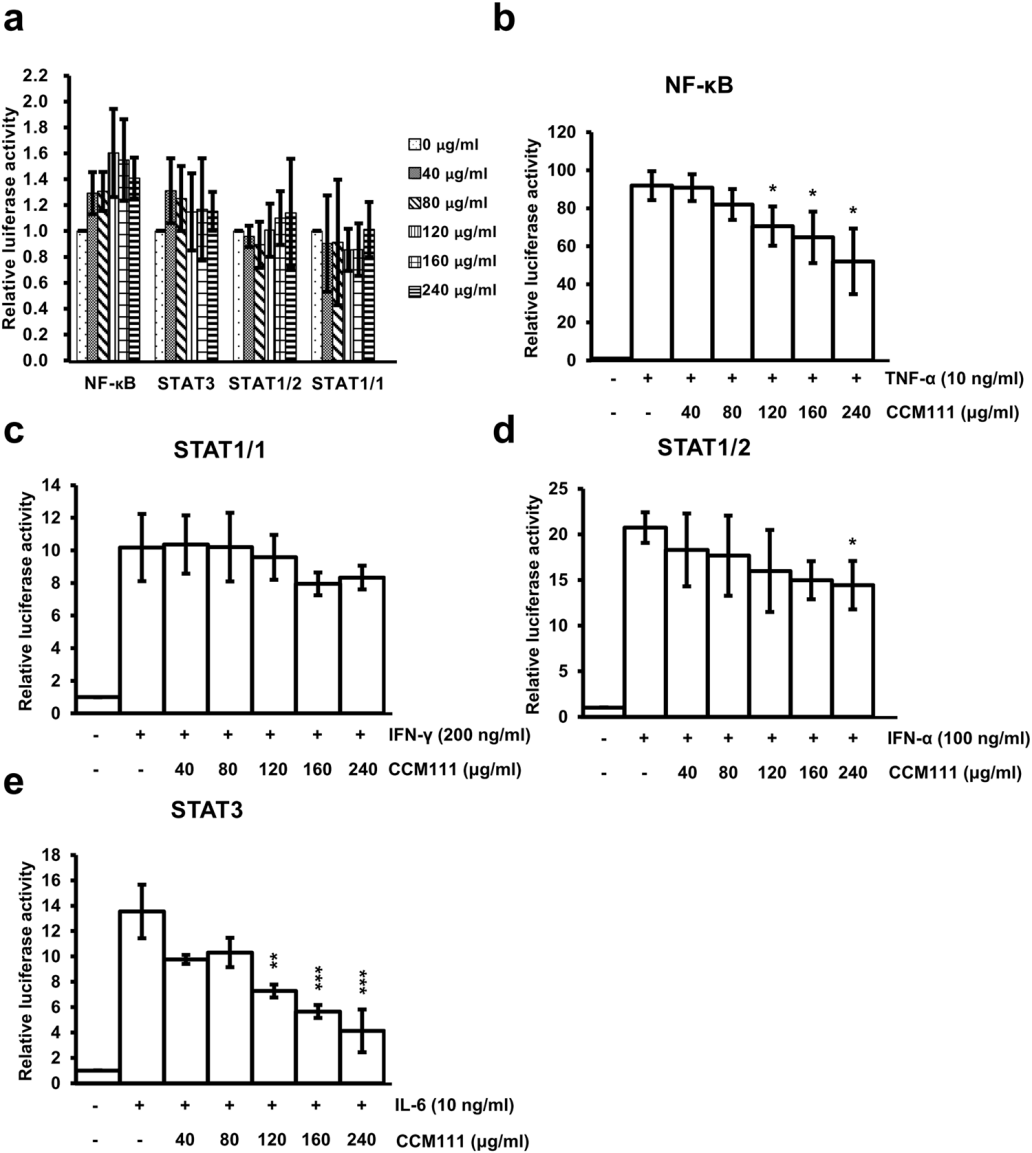
The effects of CCM111 on immune signaling pathways. Four stable cell lines expressing STAT1/1-, STAT1/2-, STAT3- or NF-κB-dependent luciferase reporters were treated with different concentrations of CCM111 (0, 40, 80, 120, 160 and 240 μg/ml) as shown in (**a**). The STAT1/2, STAT3 and NF-κB reporters were constructed in the HEK293 cell line. The STAT1/1 was constructed in the HeLa cell line. Cells were treated with individual ligands alone or in combination with different doses of CCM111 as shown in (**b**–**e**). The luciferase activity of the NF-κB was detected at 4 hours, and the luciferase activities of STAT1/1, STAT1/2 and STAT3 were measured at 17 hours. The S.D. was performed by Student’s *t*-test compared to the individual ligand group. *p-value < 0.05, **p-value < 0.01 and ***p-value < 0.001. Results were obtained from three independent replicates.

To explore the role of CCM111 in the immune system, we generated a screening platform to detect immune-related signal transduction pathways in cells. We established stable cells containing signaling-dependent transcriptional regulatory elements placed in front of luciferase reporters. First, we investigated if CCM111 influenced the STAT1/1 pathway in HeLa cells and the STAT1/2, STAT3 and NF-κB pathways in HEK293 cells (Fig. 2). CCM111 treatment alone slightly activated the NF-κB signaling pathway, but it did not affect the STAT1/1, STAT1/2 and STAT3 signaling pathways (Fig. 2a). We also investigated the effects of CCM111 after activation of these signaling pathways by stimulating the transcriptional activity of NF-κB, STAT1/1, STAT1/2 and STAT3 by TNF-α, IFN-γ, IFN-α and IL-6, respectively (Fig. 2b-e). The reporter activity for STAT1/2 stimulated by IFN-α was significantly reduced at 240 μg/ml, but the reporter activity of STAT1/1 stimulated by IFN-γ was not affected (Fig. 2c,d). The IL-6-induced reporter activity of STAT3 and the TNF-α-induced NF-κB activity were decreased significantly after CCM111 treatment in a dose-dependent manner (Fig. 2b,e). However, the inhibition effects of CCM111 toward the NF-κB pathway decreased over time (Fig. 2b and Supplementary Fig. 1b). Previous studies have shown that the anti-inflammatory activity of AC occurs mainly through inhibition of the NF-κB signaling pathway^37^. Interestingly, we found that CCM111 inhibited the STAT3 signaling pathway, which also plays a crucial role in inflammation.

### Effects of CCM111 on the NF-κB and STAT3 pathways in HEK293 cells

**Figure 3:**
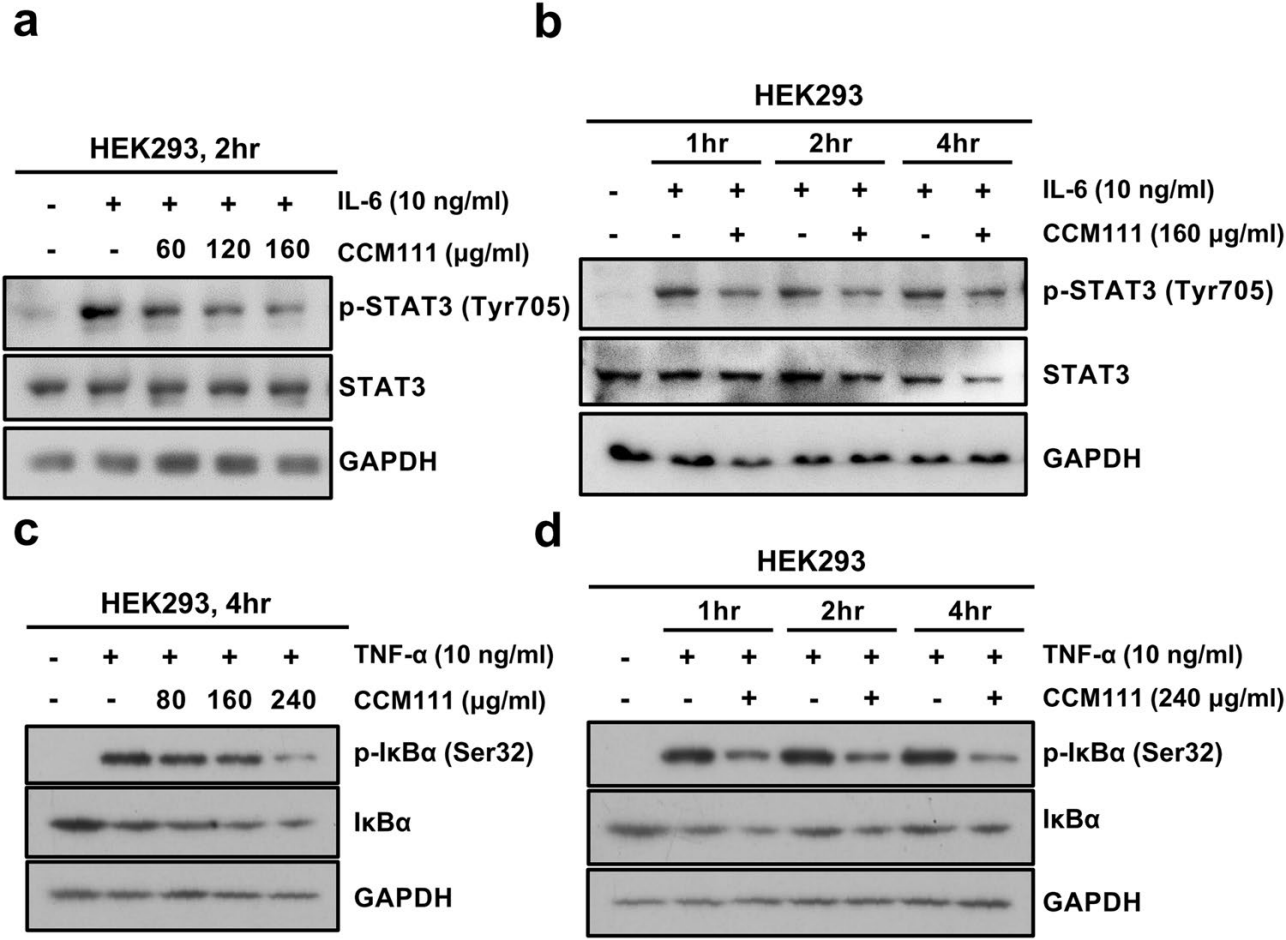
The effects of CCM111 on the STAT3 signaling transduction pathway in HEK293 cells. (**a**–**d**) Total cell lysates were prepared for Western blot analysis to detect the protein levels. (**a**) The cells were treated with h-IL-6 (10 ng/ml) and CCM111 (0, 60, 120 or 160 μg/ml) for 2 hours. (**b**) The cells were treated with h-IL-6 (10 ng/ml) alone or in combination with CCM111 (160 μg/ml) for 1, 2 and 4 hours. (**c**) The cells were treated with TNF-α (10 ng/ml) and CCM111 (0, 80, 160 or 240 μg/ml) for 2 hours. (**d**) The cells were treated with TNF-α (10 ng/ml) alone and/or in combination with CCM111 (240 μg/ml) for 1, 2 and 4 hours. (**a**,**b**) The protein levels of STAT3 and phospho-STAT3 were detected. (**c**,**d**) The protein levels of IκBα and phospho-IκBα were detected. GAPDH served as an internal control. The full length blots images are presented in Supplementary Fig. 2. Results were obtained from three independent replicates.

To clarify the effects of CCM111 on the STAT3 and NF-κB signaling pathways, we verified the reporter signaling results by directly detecting the phosphorylation status of STAT3 and IκBα. As shown in Fig. 3, CCM111 significantly inhibited IL-6-mediated phosphorylation of STAT3 at Tyr705 in a dose- and time-dependent manner (Fig. 3a,b). CCM111 at a higher concentration (240 μg/ml) suppressed TNF-α-induced phosphorylation of IκBα at Ser32, resulting in protein degradation (Fig. 3c,d). The results suggested that CCM111 may suppress the STAT3 and NF-κB signaling pathways in HEK293 cell line.

### Effects of CCM111 on the TLR2, TLR3 and TLR4 pathways in HEK293 cells

**Figure 4:**
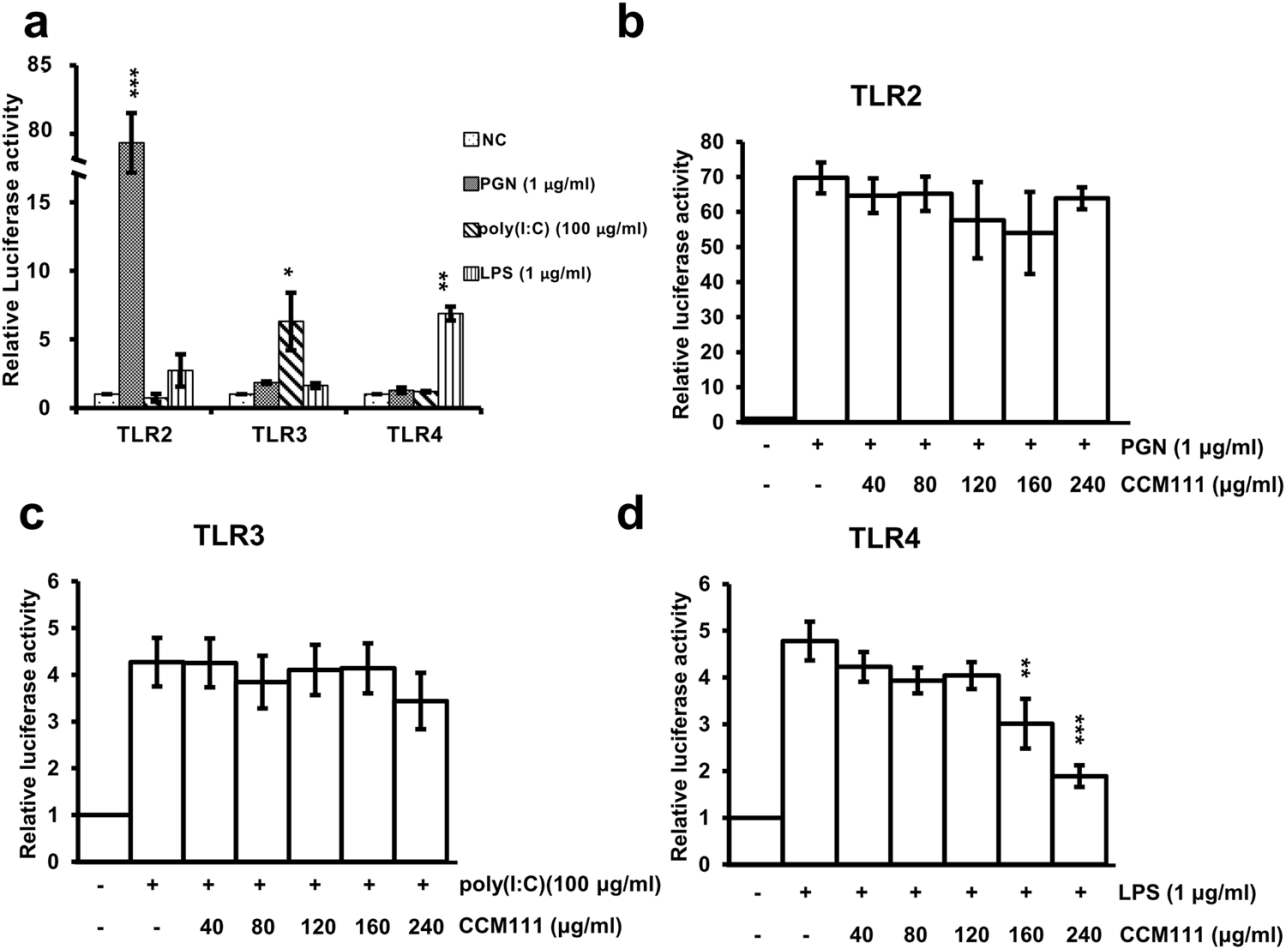
The effects of CCM111 on TLR signaling pathways. Three stable cell lines separately expressing TLR2-, TLR3- or TLR4-dependent luciferase reporters were separately treated with LPS (1 μg/ml), PGN (1 μg/ml), and poly(I:C) (100 μg/ml) for 17 hours as shown in (**a**). TLR2, TLR3 and TLR4 were constructed in the HEK293 cell line. Cells were treated with individual ligands and different doses of CCM111 as shown in (**b**–**d**). After 17 hours, the luciferase activity was measured. The S.D. was performed by Student’s *t*-test compared to the individual ligand group. **p-value < 0.01 and ***p-value < 0.001. Results were obtained from three independent replicates.

To investigate the effects of CCM111 on infection-mediated signaling, we further investigated the effects of CCM111 on the TLR2, TLR3 and TLR4 signaling pathways using stable cells and luciferase reporter assays. We stimulated the TLR2, TLR3 and TLR4 signaling pathways by PGN, poly(I:C), and LPS to mimic gram-positive bacteria, RNA viruses, and gram-negative bacteria, respectively, to measure the NF-κB luciferase reporter element responses in HEK293 cells. First, we treated the reporter clones with different ligands to demonstrate the specificity of three TLR luciferase reporters (Fig. 4a). We observed that treatment with CCM111 did not influence TLR2 and TLR3 signaling but significantly decreased TLR4 signaling at higher concentrations (160 and 240 μg/ml) (Fig. 4d). TLR4 is suggested to be upstream of NF-κB and STAT3, indicating that these data were consistent with our previous reporter results in Fig. 2 ^28^. These results suggested that CCM111 could protect against the immune responses of gram-negative bacterial infections through the NF-κB and STAT3 pathways.

### Effects of CCM111 on LPS-induced STAT3 and NF-κB signaling in macrophage cells

**Figure 5:**
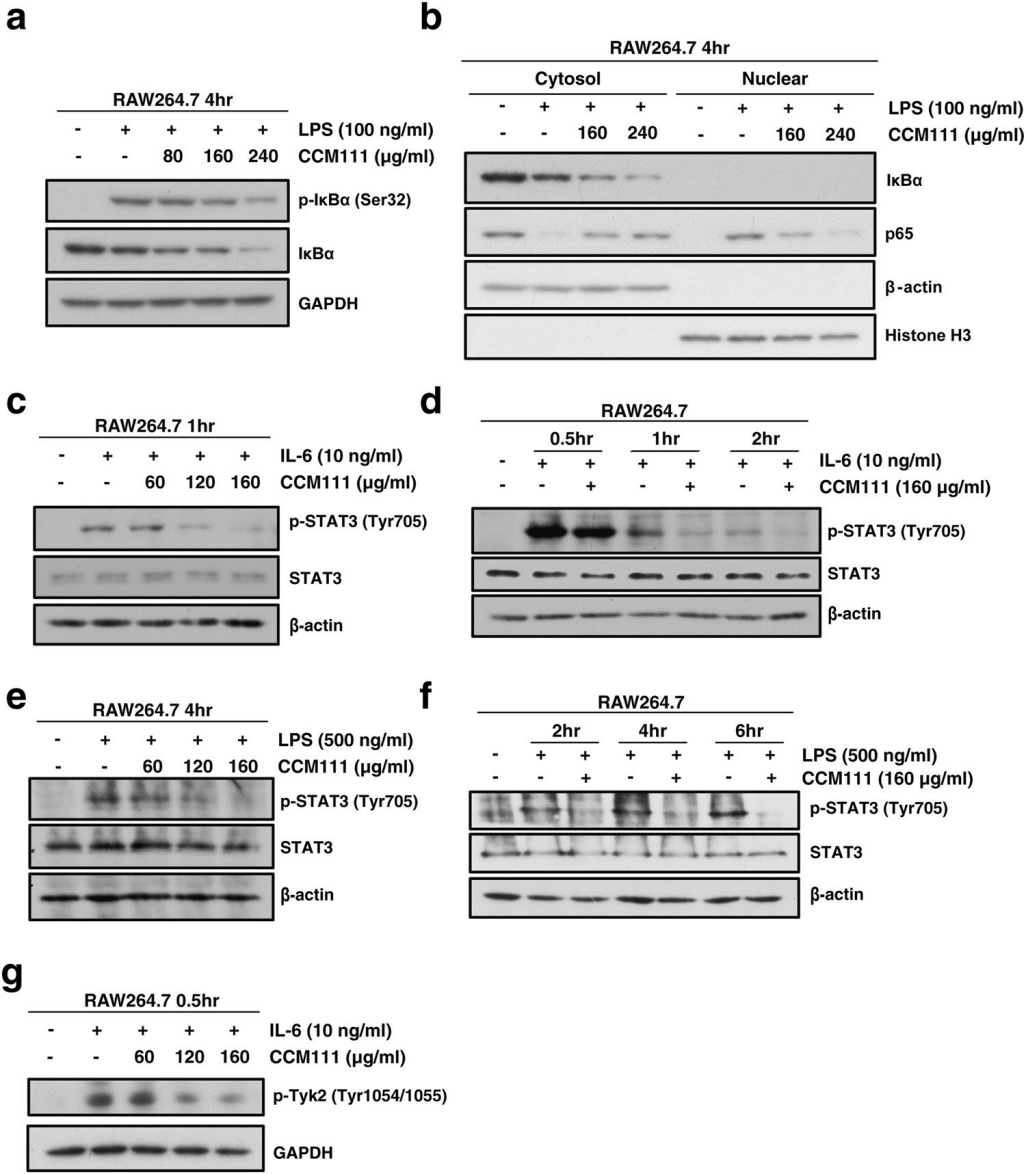
The effects of CCM111 on NF-κB and STAT3 pathway activities in murine RAW264.7 macrophages. (**a**) The cells were treated with LPS (100 ng/ml) and CCM111 (0, 80, 160 or 240 μg/ml) for 4 hours. (**b**) The cells were treated with 100 ng/mL LPS alone or with different concentrations of CCM111 (0, 160 and 240 μg/ml) for 4 h. (**c**) The cells were treated with IL-6 (10 ng/ml) and CCM111 (0, 60, 120 or 160 μg/ml) for 1 hour. (**d**) The cells were treated with IL-6 (10 ng/ml) alone or in combination with CCM111 (160 μg/ml) for 0.5, 1, and 2 hours. (**e**) The cells were treated with LPS (500 ng/ml) and CCM111 (0, 80, 160 or 160 μg/ml) for 4 hours. (**f**) The cells were treated with LPS (500 ng/ml) alone or in combination with CCM111 (160 μg/ml) for 2, 4, and 6 hours. (**g**) The cells were treated with IL-6 (10 ng/ml) alone or in combination with CCM111 (0, 60, 120, 160 μg/ml) for 0.5 hours. (**a**–**g**) After treatment, the cell lysates were analyzed by immunoblotting. (**a**) The protein levels of IκBα and phospho-IκBα were detected, and GAPDH was used as the internal control. (**b**) The protein levels of p65, IκBα expression were detected. β-actin was used as the cytosol internal control, and Histone H3 was used as the nuclear internal control. (**c**–**f**) The protein levels of STAT3 and phospho-STAT3 were detected, and β-actin was used as the internal control. (**g**) The protein level of phospho-Tyk2 was detected, and GAPDH was used as the internal control. The full length blots images are presented in Supplementary Fig. 3. The results were obtained from three independent replicates.

We demonstrated that CCM111 influences both the IL-6-mediated STAT3 signaling pathway and the LPS-induced NF-κB signaling pathway according to luciferase assays using HEK293 cells. These two pathways trigger inflammatory cytokine expression in macrophage cells^2^. Therefore, we investigated if CCM111 affects the mechanism of the STAT3 and NF-κB pathways in RAW264.7 macrophages. Interestingly, although CCM111 reduced the LPS-induced phosphorylation of IκBα, we found that CCM111 also decreased the IκBα protein expression in RAW264.7 cells (Fig. 5a). Therefore, we further examined if NF-κB translocation will be regulated by CCM111. This result suggested that CCM111 reduced the activity of NF-κB pathway activity through blocking the translocation of p65 in RAW264.7 cells (Fig. 5b). Next, we checked if CCM111 can affect the IL-6-mediated phosphorylation of STAT3 (Fig. 5c,d). As consistent with previous results, CCM111 reduced the IL-6-mediated phosphorylation of STAT3 in RAW264.7 cells. We further investigated the mechanism of CCM111 in the regulation of STAT3 signaling because LPS can induce STAT3 activation in macrophages^40^. CCM111 decreased the phosphorylation of STAT3 induced by LPS in a dose- and time-dependent manner (Fig. 5e,f). These results suggested that CCM111 suppressed STAT3 and NF-κB pathway activity by blocking the phosphorylation of STAT3 and the nuclear translocation of p65 to further inhibit the downstream transcriptional activity in macrophages.

To further investigate the specific mechanism of CCM111 on the STAT3 rather than the STAT1/1 pathway in macrophages (Fig. 2c,d), we detected the phosphorylation status of Tyk2, with this specific kinase chosen because STAT3 signaling activation requires phosphorylation of Tyk2^41^. CCM111 significantly inhibited the IL-6-mediated phosphorylation of Tyk2 at Tyr1054/1055 in a dose-dependent manner (Fig. 5g). The results showed that CCM111 inhibition of the STAT3 pathway activity may act through reduction of the phosphorylation of Tyk2 protein expression.

### Effects of CCM111 on the expression of inflammatory cytokines in RAW264.7 cells

**Figure 6:**
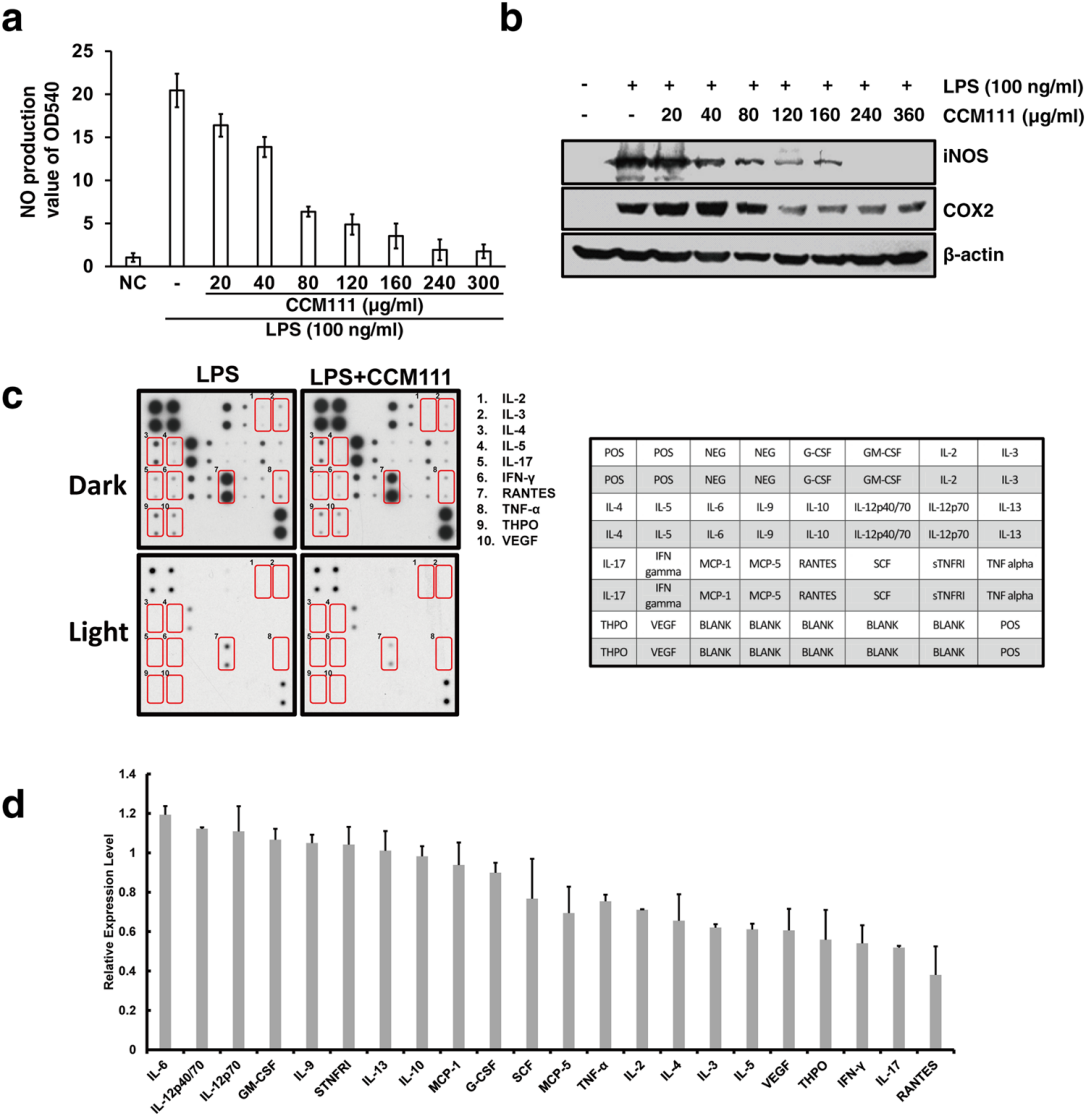
The effects of CCM111 on NO production and the protein expression of iNOS, COX-2, and inflammatory cytokines in murine RAW264.7 macrophages. The cells were treated with LPS (100 ng/ml) alone or in combination with different concentrations of CCM111. After incubation for 24 hours, the culture media was collected for nitrate assay analysis as shown in (**a**), and the protein lysates were analyzed by immunoblotting for iNOS, COX2 and β-actin as shown in (**b**) The full length blots images are presented in Supplementary Fig. 5a. Results in (**a**) and (**b**) were obtained from three independent replicates. The cells were treated with LPS (100 ng/ml) alone or in combination with LPS and CCM111 (160 μg/ml) for 24 h. The cell lysates were analyzed by the RayBio C-series mouse cytokine antibody array C1. (**c**) The left panel shows an image of the signal spots on the membrane for each cytokine. Each spot represents one cytokine, and each cytokine is in duplicate. Spots that show significant change are marked. The right panel shows the identities of all spots on the array with coordinates. The full length blots images are presented in Supplementary Fig. 5b. The left image of spots was scanned and measured by NIH Image-J software. (**d**) The relative expression level of each protein was calculated from densitometry data from (**c**) and normalized to the LPS treatment alone array. The blue dotted line represents a 0.8-fold difference, and the red dotted line represents a 1.2-fold difference. Error bars indicate S.D. (n = 2).

Because CCM111 treatment suppressed the STAT3 and NF-κB signaling pathways, we next examined the effects of CCM111 on their downstream targets. The STAT3 and NF-κB signaling pathways regulate pro-inflammatory cytokines, including iNOS and COX-2 expression, as well as NO production^8, 16^. Therefore, to test the inflammatory effects of CCM111 in macrophage cells, we measured LPS-induced NO production after CCM111 pretreatment. LPS alone significantly increased nitrate production, but CCM111 potently inhibited LPS-induced nitrite production in a dose-dependent manner (Fig. 6a). Interestingly, we used the STAT3 pathway inhibitor, static, which could reduce LPS-induced NO production in RAW264.7. This result suggested that NO production was dependent on STAT3 (Supplementary Fig. 4). Moreover, the increased protein expression of iNOS and COX-2 was inhibited by CCM111 in a dose-dependent manner (Fig. 6b).

Macrophages can produce cytokines to regulate lymphocytes in inflammatory responses. Using the mouse cytokine antibody array, we next examined if CCM111 could reduce the production of inflammatory cytokines in addition to iNOS and COX-2 in LPS-stimulated RAW264.7 macrophages (Fig. 6c). Among the 22 tested cytokines, CCM111 down-regulated the production of the following 10 LPS-induced cytokines: TNF-α, IL-2, IL-4, IL-3, IL-5, VEGF, THPO, IFN-γ, IL-17, and RANTE. However, other pro-inflammatory cytokines, such as IL-6, IL-12p40/70, IL-12p70, and IL-4, were not altered by CCM111 treatment (Fig. 6d). The pro-inflammatory cytokines, such as TNF-α, IL-4 and IFN-γ, are important mediators in inflammatory responses, and the protein levels of these cytokines were suppressed by CCM111 treatment. This result implied that CCM111 reduced inflammation responses through specific pathways.

### Effects of CCM111 fractions on the NO production and the phosphorylation of Tyk2 protein expression in RAW264.7 cells

**Figure 7:**
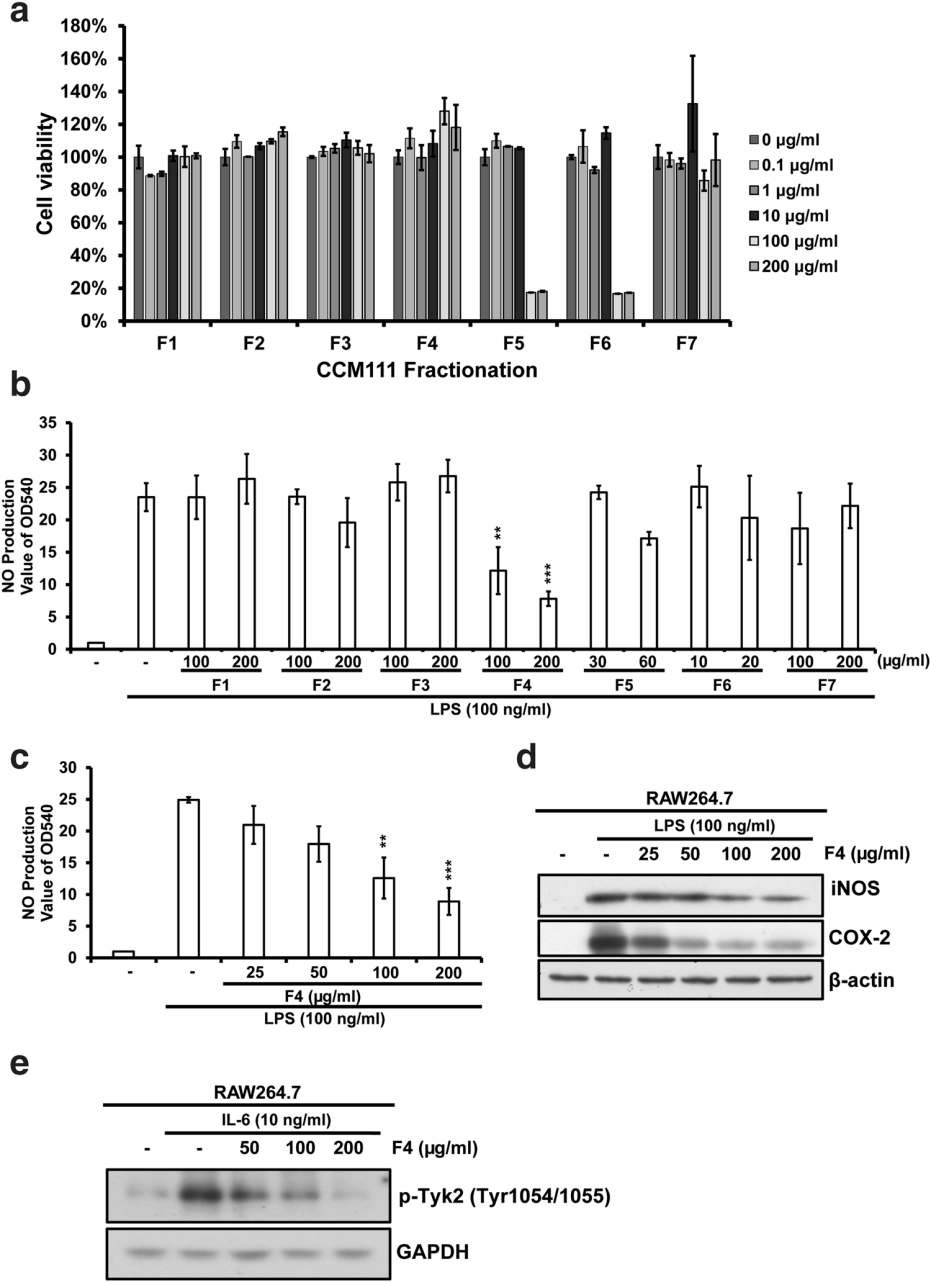
The effects of CCM111 fractions on NO production and the protein expressions of iNOS, COX-2 in murine RAW264.7 macrophages. (**a**) RAW264.7 cells were treated separately with different concentrations of CCM111 fractions for 24 hours. (**b**) The cells were treated with LPS (100 ng/ml) alone or in combination with different concentrations of CCM111 fractions. (**c**,**d**) The cells were treated with LPS (100 ng/ml) alone or in combination with different concentrations of fraction 4 for 24 hours. (**e**) The cells were treated with IL-6 (10ng/ml) alone or in combination with F4 for 0.5 hours. (**a**) After treatment, the survival of cells was detected by the Alamar Blue assay. (**b**,**c**) After incubation, the culture media were collected for nitrate assay analysis. (**d**,**e**) After treatment, the cell lysates were analyzed by immunoblotting. (**d**) The protein levels of iNOS and COX-2 were detected, and β-actin was used as the internal control. (**e**) The protein level of phospho-Tyk2 was detected, and GAPDH was used as the internal control. The full length blots images are presented in Supplementary Fig. 6. The S.D. was performed by Student’s *t*-test compared to the control or LPS only group. **p-value < 0.01 and ***p-value < 0.001. Results were obtained from three independent replicates.

To examine possible components for the anti-inflammatory activity of CCM111, we further chromatographed CCM111 over Dianion HP-20, eluted with acetone and gradients of H_2_O/EtOH (100:0, 80:20, 60:40, 40:60, 20:80, 5:95), and 7 fractions (F1–F7) were collected. We performed the toxicity tests for these fractions in RAW 264.7 macrophages. F5 and F6 at 100 and 200 μg/ml could induce cell toxicity after treatment for 24 hours. (Fig. 7a). We further examined which fractions demonstrated anti-inflammatory activity in RAW 264.7 macrophages. Fraction 4 (F4) significantly reduced the LPS-induced nitrite production in a dose-dependent manner (Fig. 7b-c). Moreover, the increased protein expression of iNOS and COX-2 was inhibited by F4 in a dose-dependent manner (Fig. 7d). Interestingly, the IL-6-induced phosphorylation of Tyk2 was also inhibited by F4 treatment (Fig. 7e). F4 was analyzed by LC/MS analysis and presented at least six peaks (peaks 1–6) (Supplementary Fig. 7). The results implied that F4 is the major fraction for anti-inflammatory activity.

## Discussion

**Figure 8:**
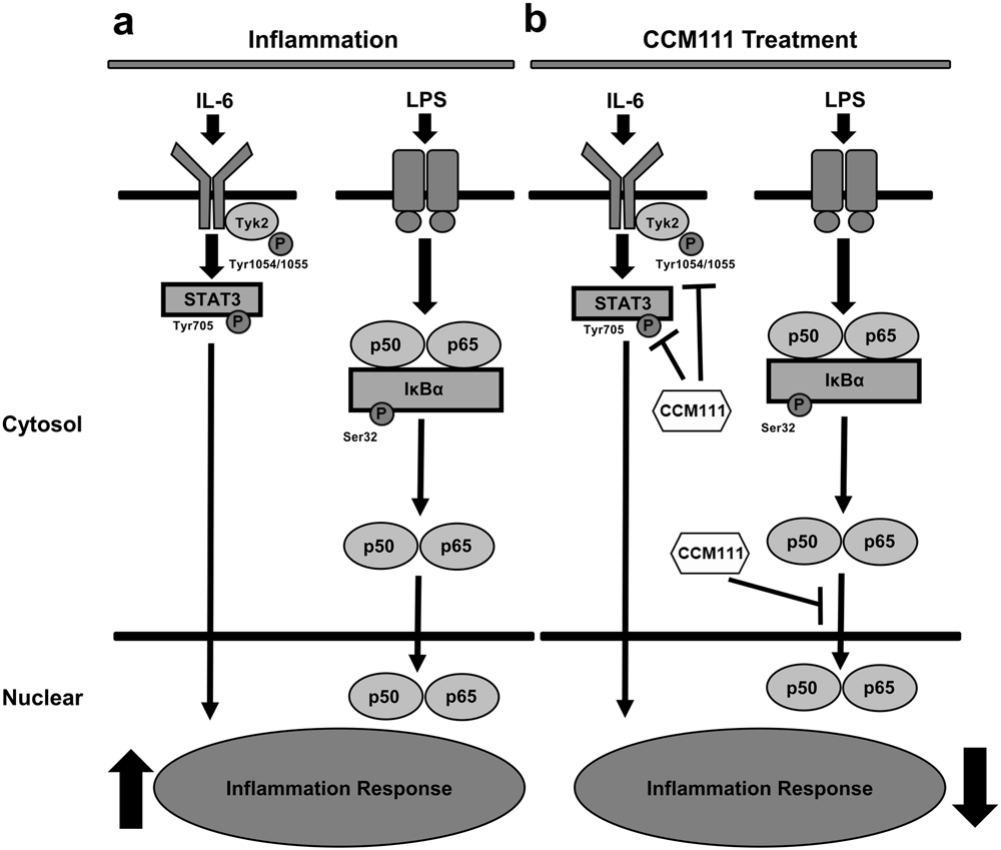
The model of effects of CCM111 in macrophage cells. (**a**) LPS induces the activation of the TLR4/NF-κB pathway and up-regulates IL-6 to activate the STAT3 pathway. The activation of the STAT3 and NF-κB pathways results in the enhancement of inflammatory responses. (**b**) CCM111 significantly suppresses the STAT3 pathway through inhibiting the phosphorylation of STAT3 and Tyk2, and it suppresses the NF-κB pathway by inhibiting the nuclear translocation of p65 to repress LPS-induced inflammatory responses.

We screened the effects of CCM111, the water extract of AC, on the modulation of inflammatory signaling pathways, and we elucidated the effects and mechanisms of CCM111 on LPS-induced murine macrophages. We also provided molecular evidence for CCM111 serving as a potential anti-inflammatory drug. Previous studies have suggested that the anti-inflammatory activity of AC occurs through inhibition of the NF-κB pathway^42^. Our results demonstrated that CCM111 significantly reduces the NF-κB and STAT3 signaling pathways (Figs 2 and 5). Moreover, CCM111 can reduce pro-inflammatory cytokines in LPS-induced murine macrophages. Our current proposed model suggests that inflammatory responses are triggered when LPS induces the activation of the TLR4/NF-κB pathways, which produce IL-6 to activate the STAT3 pathway (Fig. 8a). CCM111 inhibits the phosphorylation of STAT3, Tyk2 and the nuclear translocation of p65 to reduce inflammatory responses (Fig. 8b).

AC is commonly used as a functional hepatoprotective or immune-regulation herb in Taiwan^30^. However, we demonstrated that CCM111 had little effect on the activation status of the immune-related pathways of STAT1/1, STAT1/2, STAT3, and NF-κB under no stimulation. These results indicated that CCM111 may not affect the immune system in normal conditions (Fig. 2a). In our study, only treatment with IFN-α markedly increased the activity of STAT1/2 pathway, and CCM111 significantly decreased the phosphorylation of STAT1/2 at 240 μg/ml in HEK293 cells (Fig. 2d). In addition, CCM111 and F4 inhibited the phosphorylation of Tyk2 upon IL-6 in macrophage cells (Figs 5g and 7e). Tyk2 is one of the JAK kinases and regulates the activation of the STAT1/2 and STAT3 pathways^43^. These results suggested that CCM111 may inhibit STAT3 and STAT1/2 pathway activity by reducing the phosphorylation of Tyk2 (Fig. 2d and e). STAT3 is involved in the response of LPS-induced IL-1β production in macrophages, and it is partly dependent on the phosphorylation at tyrosine 705^44^. In our study, we showed that treatment with LPS or IL-6 alone markedly increased the phosphorylation of STAT3 at Tyr 705 and that CCM111 robustly decreased the phosphorylation of STAT3 at Tyr 705 in a dose- and time-dependent manner in macrophage cells or epithelial cells (Figs 3a,b and 5e,f). CCM111 at high concentrations (160 and 240 μg/ml) reduced the TLR4 pathway activity significantly (Fig. 4d). This result may link with the previous findings about AC. Zhankuic acid A (ZAA), a compound of AC, is a competitive antagonist at the TLR4 receptor^45^. Overproduction of NO by iNOS is involved in different inflammatory diseases and tumorigenesis. Thus, iNOS is considered to be a potential target of anti-inflammatory activity. There are various binding sites for transcription factors, including NF-κB and STATs, in the iNOS promoter region^46^. Taken together, these data are consistent with our results, showing that CCM111 and F4 inhibit LPS-induced NO production and iNOS expression (Figs 6a,b and 7c,d).

We showed that CCM111 reduced LPS-stimulated secretion of proteins, including TNF-α, IL-2, IL-4, IL3, IL-5, VEGF, THPO, IL-17 and RANTE, which are pro-inflammatory cytokines and chemokines (Fig. 6c,d). RANTE recruits immune cells to inflammation and infection sites, and it is involved in the activation of the adaptive immune response^47^. TNF-α is a key mediator of inflammation, vascular permeability and cytokine production, and it activates the adaptive immune response through contributing to the proliferative response in T lymphocytes^48, 49^. IL-2 is a critical regulator of group 2 innate lymphoid cell function during pulmonary inflammation^50^, and IL-3 is considered to be the therapeutic marker of septic inflammation^51^. IL-5 is a cytokine that induces differentiation of B cells and promotes the proliferation and differentiation of eosinophils into mature eosinophils in humans^52^. IL-17A mainly mediates its immune regulatory function by promoting the generation of pro-inflammatory cytokines and chemokines, which leads to the attraction of neutrophils and macrophages to the inflammation site^53^. The pro-inflammatory action of IL-17 depends considerably on its ability to trigger the expression of iNOS^54^. Interferon-γ is a pro-inflammatory cytokine produced by macrophages and is important in early immune defense^55^. THPO can regulate neutrophil motility and mobilization as well as the differentiation and proliferation of megakaryocyte progenitors^56, 57^. VEGF is the marker for angiogenesis regulation of inflammation^58^, and the expression of VEGF is regulated by NF-κB^59^. Therefore, upon LPS treatment, the decrease in these pro-inflammatory cytokines and chemokines by CCM111 indicates that CCM111 may prevent the recruitment of immune cells, thus limiting the host inflammatory and immune responses. However, CCM111 also reduces IL-4, which can induce the peroxisome proliferator-activated receptor-γ (PPARγ) transcription factor and is involved in suppressing inflammation in macrophages^60^. Interestingly, IL-4 promotes M2 macrophage formation, which is associated with chronic inflammation^61^, thereby suggesting that CCM111 may alleviate chronic inflammation. In addition, CCM111 had no effects on the secretion of IL-6 and IL-12p40p70 or the IL-10 anti-inflammatory cytokine (Fig. 6c,d), thus indicating that CCM111 is involved in specific signal transduction pathways as shown in this study.

In summary, our study provided novel insight into the mechanism of AC in signaling pathways. Our results suggested that CCM111 reduces the TLR4, NF-κB and STAT3 signaling pathways, which function to regulate immune responses. CCM111 has anti-inflammatory effects and decreases LPS-induced iNOS and COX-2 expression in macrophage cells by affecting the phosphorylation of STAT3, Tyk2 and the nuclear translocation of p65. Based on these findings, we suggest that CCM111 has potential as an anti-inflammatory agent for the treatment of a variety of inflammatory diseases.

## Materials and Methods

### Chemicals and reagents

Interferon-alpha (IFN-α) and peptidoglycan (PGN) were purchased from Sigma-Aldrich (Sigma-Aldrich, MO, USA). Tumor necrosis factor alpha (TNF-α), interferon gamma (IFN-γ), human interleukin 6 (h-IL-6), Phorbol-ester 12-o-tetradecanoyl phorbol 13-acetate (TPA), lipopolysaccharide (LPS) and polyinosinic-polycytidylic acid (poly(I:C)) were obtained from PeproTech (Rocky Hill, NJ, USA). Stattic was obtained from TargetMol (TargetMol, MA, USA). *Antrodia cinnamomea* was provided by the Brion Institute of Taiwan. Methanol, ethanol, phosphoric acid, acetonitrile were obtained from Sigma-Aldrich (Sigma-Aldrich, MO, USA).

### Preparation of the crude water extract

The *Antrodia cinnamomea* mycelia culture broth was concentrated under vacuum and freeze-dried to a powder form. For the preparation of the aqueous solution, the powder samples were solubilized with sterilized water at 80 °C for 30 min and then centrifuged for 10 min at 10,000 rpm after passage through a 0.2 μm pore-size filter. The stock solution was stored at −20 °C before analysis.

### Cell lines and establishment of stable cell lines

HEK293 and HeLa cells were obtained from American Type Culture Collection (ATCC, VA, USA). RAW264.7 cells were purchased from the Food Industry Research and Development Institute (Hsinchu, Taiwan). The growth medium used for HEK293, HeLa and RAW 264.7 cells was Dulbecco’s Modified Eagle Medium (Gibco Life Technologies, Grand Island, NY, USA) with 10% heat-inactivated fetal bovine serum (Biological Industries, SC, USA), 1 mM L-glutamate and 1 mM penicillin/streptomycin. All of the cell lines were incubated at 37 °C with 5% carbon dioxide. The cells were plated at approximately 60–70% confluency in a 12-well plate. The following day, 400 ng of plasmid DNA, 50 µl of Opti-MEM and 1.5 µl of FuGENE HD (Roche, Mannhein, Germany) were mixed and incubated at room temperature for 15 min. The transfection complex was then added to the cells. After 24 hours, cells were subcultured into 510-cm dishes and incubated for an additional 48 hours. Stable cells lines were generated by culturing in selection media containing 0.2 μg/ml puromycin. Individual clones were picked and transferred to 96-well plates after 2–3 weeks of puromycin selection.

### Establishment of stable clones expressing the transcriptional response element (TRE) luciferase reporter

Transcriptional regulatory elements (TREs) are the transcription factor binding sequences. The TRE forward primer (50 mM) was annealed with the TRE reverse primer (50 mM). The annealed TRE sequence was ligated into the promoter region of the pGL4.20 vector containing a luciferase reporter gene (Promega, WI, USA). Three tandem repeats of consensus TRE sequence were inserted into the NheI-BglII site of pGL4. The TRE sequences used in this experiment are described in Supplementary Table 1. After cloning and sequencing the plasmid, the reporter plasmid was separately transfected into HEK-293 or HeLa cells using FuGENE HD (Roche, Mannheim, Germany). After transfection, the cloning was selected by puromycin (0.2 μg/ml). The STAT1/2, STAT3, TLR2, TLR3, TLR4, and NF-κB luciferase reporter clones were constructed in HEK293, and STAT1/1 was constructed in the HeLa cell line. Prof. Yung-Chi Cheng at Yale University kindly provided the 6 stable cell lines expressing the TLR2-, TLR3-, TLR4-, NF-κB-, Stat1/2- and Stat3-responsive luciferase reporters.

### Luciferase reporter assay

The stable clonal cell lines were separately seeded at 2.5 × 10^4^ cells/well in 96-well plates. After 24 hours, specific ligands that induce the signal transduction pathways were then added to the wells. CCM111 was added to cells at concentrations ranging from 0 to 120 μg/ml. After 18 hours, the medium was removed. Lysis buffer was then added, and the cells were placed in an orbital shaker for 30 min for complete lysis. Lysed cells were transferred into white 96-well plates containing luciferase buffer, and luminescence was detected at an emission wavelength of 460/40 using a Synergy HT (Biotech, VT, USA).

### Toxicity assay

A total of 2 × 10^4^ cells were plated in 96-well plates and treated with different concentrations of CCM111 (0, 20, 40, 80, 120, 240, and 360 μg/ml). After 24 hours, cell toxicity was determined by AlamarBlue assays (Invitrogen Life Technologies, NY, USA) according to the manufacturer’s instructions. The fluorescence values were measured at excitation wavelengths of 530–560 nm and an emission wavelength of 590 nm. All measured values were detected using a Synergy HT (BioTek, VT, USA).

### Western blotting assay

Cells were washed in phosphate-buffered saline (PBS), and proteins were extracted in NP-40 buffer (50 mM Tris-HCl, pH 7.5, 150 mM NaCl, 1% NP-40, 10% glycerol, and 1 mM EDTA). The cell lysates were then resolved by electrophoresis through SDS-polyacrylamide gels, and the proteins were electrotransferred onto PVDF membranes. Milk- or BSA-blocked blots were incubated with primary antibodies at 4 °C overnight and then incubated with horseradish peroxidase-conjugated (HRP) secondary antibodies (Cell Signaling Technology, Inc., MA, USA.). The proteins were detected by enhanced chemiluminescence using Western Blotting Detection Reagents (LuminataTM Classico/Forte Western HRP substrate). The primary antibodies against p-Tyk2, p-Stat3, Stat3, p-IκBα, IκBα, NF-κB p65 and Histone H3 were from Cell Signaling Technology (Cell Signaling Technology, Inc., MA, USA). The primary antibodies against COX-2 and iNOS were from the Transduction Laboratories (BD Transduction Laboratories, KY, USA). The β-actin antibody was from Oncogene Science (Oncogene Science Inc., Uniondale, NJ).

### Nitrite assay

The nitrite concentration in the culture medium was measured as an indicator of NO production. After centrifugation at 1000 g for 20 min, 100 μl of each supernatant medium was mixed with the same volume of Griess reagent (1% sulfanilamide in 5% phosphoric acid and 0.1% naphthylethylenediamine dihydrochloride in water). The absorbance of the mixture at 550 nm was measured with an enzyme-linked immunosorbent assay plate reader (Dynatech MR-7000; Dynatech Laboratories, VA, USA).

### Subcellular fractionation

The cells were suspended in 400 μl of Buffer A (10 mM HEPES pH7.9, 10 mM KCL, 0.1 mM EDTA, 1 mM DTT, and proteinase inhibitor) on ice for 15 min, and added 25 μl of 10% NP-40. The samples were vortexed for 10 seconds and centrifuged at 2000 × g for 6 min. The supernatants with cytosolic proteins were collected. The pellets inclding nuclear proteins were resuspended in 100 μl Buffer B (20 mM HEPES pH7.9, 400 mM NaCl, 1 mM EDTA, 1 mM DTT, and proteinase inhibitor proteinase inhibitor) on ice for 15 mins. The samples were sonicated 5 seconds and centrifuged 12000 × g for 5 mins. The cytosol and nuclear fractions were collected for western blot analysis.

### HPLC-UV analysis

The HPLC-UV analysis was performed using a Waters HPLC System (Milford, Massachusetts, USA) consisting of an integrated controller, a quaternary pump, a column temperature controller, an autoinjector, and a photodiode array detector. The extraction solution (100 μl) was pipetted into the vial and diluted with 5 ml of pure water. The diluent was then filtered with a 0.45 μm filter membrane and was used as the test solution. The results were obtained by linear gradient eluents A and B (A, 0.1% H_3_PO_4_; B, acetonitrile) according to the following profile: 0–10 min with 95% A and 5% B; 10–70 min with 95–50% A and 5–50% B; 70–90 min with 50–0% A and 50–100% B; 90–100 min with 0% A and 100% B; and 100–120 min with 0–95% A and 100–5% B). The following conditions were used: flow-rate of 1.0 ml/min; column temperature of 35 °C, post time of 15 min, and injection volume of 20 μl. A LiChrospher RP-18 endcapped column (5 μm, 4.0 I.D. × 10 mm, Merck, Darmstadt, Germany) was used as a guard column. A Cosmosil 5C18-MS column (5 μm, 4.6 I.D × 250 mm, Nacalai Tesque, Inc., Kyoto, Japan) was used as an analytical column.

### Antibody array

The RayBio Mouse Cytokine Array 1 (Cat. #AAM-CYT-1 RayBiotech, Inc., Norcross, GA, USA) was purchased and used to detect 22 mouse cytokines at the same time. RAW264.7 cells were seeded at 2 × 10^6^ cells/dish in 10-cm dishes. After 24 hours, the medium was changed, and the cells were incubated for 2 hours. Cells were treated with LPS (100 ng/ml) or LPS + CCM111 (160 μg/ml) for 24 hours. The cell lysate was collected, and 150 μg of total protein was run on each membrane. Kit detection buffer was added to the arrays, and the signal was detected using autoradiography film. The intensity of signals was quantified by ImageJ v1.49i Windows (National Institutes of Health, Bethesda, MD, http://rsb.info.nih.gov/ij/).

## Electronic supplementary material

Supplementary information
