# Supplementary material for: CCM111, the water extract of Antrodia cinnamomea, regulates immune-related activity through STAT3 and NF-κB pathways
Source: Sci Rep. 2017 Jul 7;7:4862. doi: 10.1038/s41598-017-05072-y (PMC5501807; doi:10.1038/s41598-017-05072-y)
Supplement: Supplementary file 1 — Supplementary information [file 41598_2017_5072_MOESM1_ESM.pdf]

SUPPLEMENTARY INFORMATION

Title: CCM111, the water extract of Antrodia cinnamomea, regulates immune-related activity through STAT3 and NF-κB pathways

Authors: In-Yu Lin, Min-Hsiung Pan, Ching-Shu Lai, Ting Ting Lin, Chiung-Tong Chen, Tao-Sheng Chung, Chien-Lung Chen, Chen-Huan Lin, Wu-Chang Chuang, Ming-Chung Lee, Ching-Che Lin, Nianhan Ma.

Pathway	TRE sequence
STAT1/1	AGTTTCATATTACTCTAAATC
STAT1/2	TAGTTTCACTTTCCC
STAT3	GTCGACATTTCCCGTAAATCGTCGA
NFκB/TLR2/ TLR3/TLR4	GGGACTTTCC

Supplementary Table 1

Transcriptional regulatory elements (TRE) are the transcription factor binding sequence. The individual TRE sequence for each of the pathway is shown in the table.

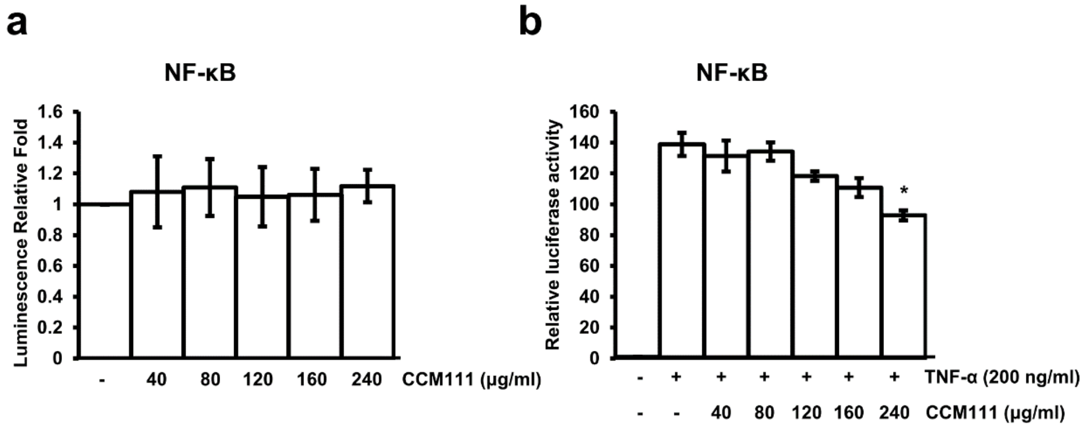

Supplementary Fig. 1

The effects of CCM111 on NF-κB-pathways. (a) NF-κB-dependent luciferase reporter was treated with different concentrations of CCM111 (0, 40, 80, 120, 160 and 240 μg/ml) for 17 hours. (b) Cells were treated with TNF-α (200 ng/ml) alone or in combination with different doses of CCM111 for 17 hours. After 17 hours, the luciferase activity was measured. The S.D. was performed by Student's *t*-test compared to the individual ligand group. *p-value < 0.05, **p-value < 0.01 and *** p-value < 0.001. Results were obtained from three independent replicates.

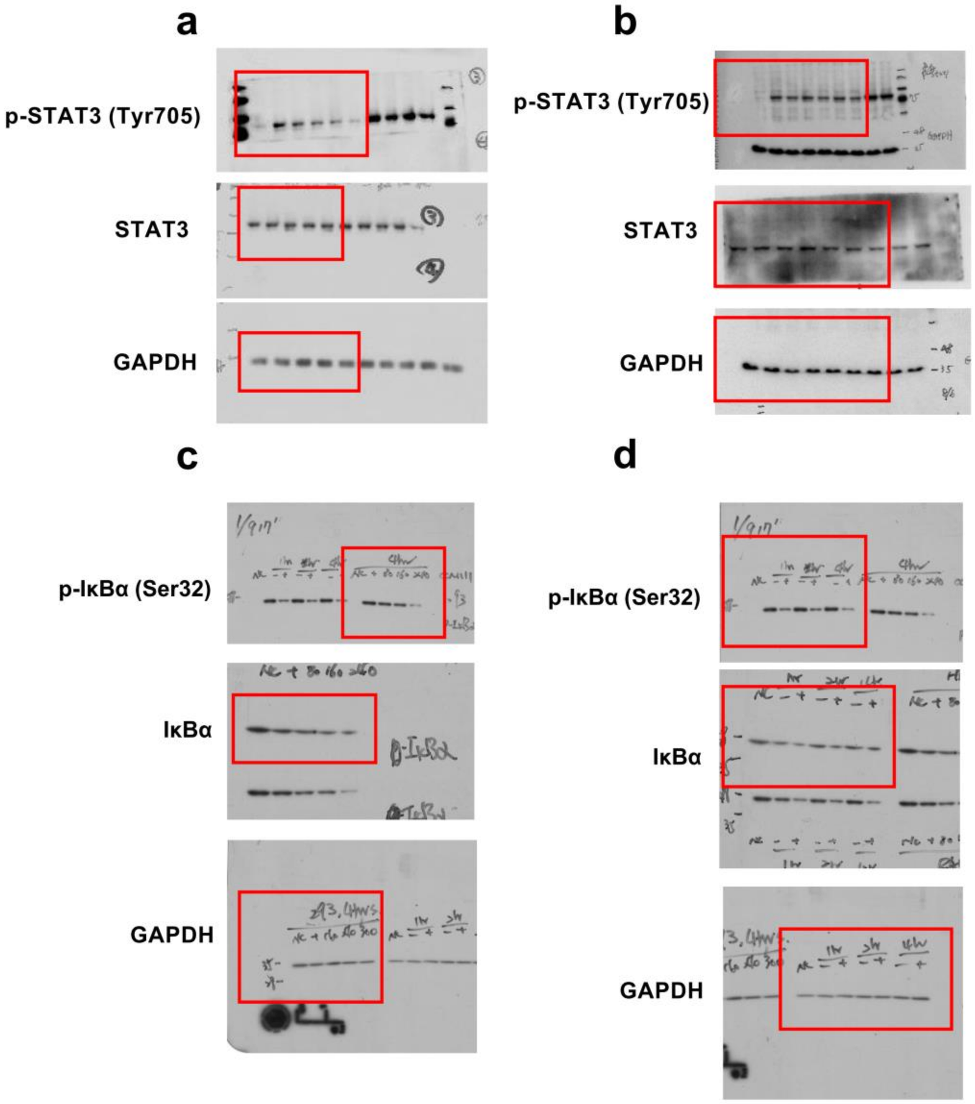

Supplementary Fig. 2

Full-length Western blot images in Fig.3.

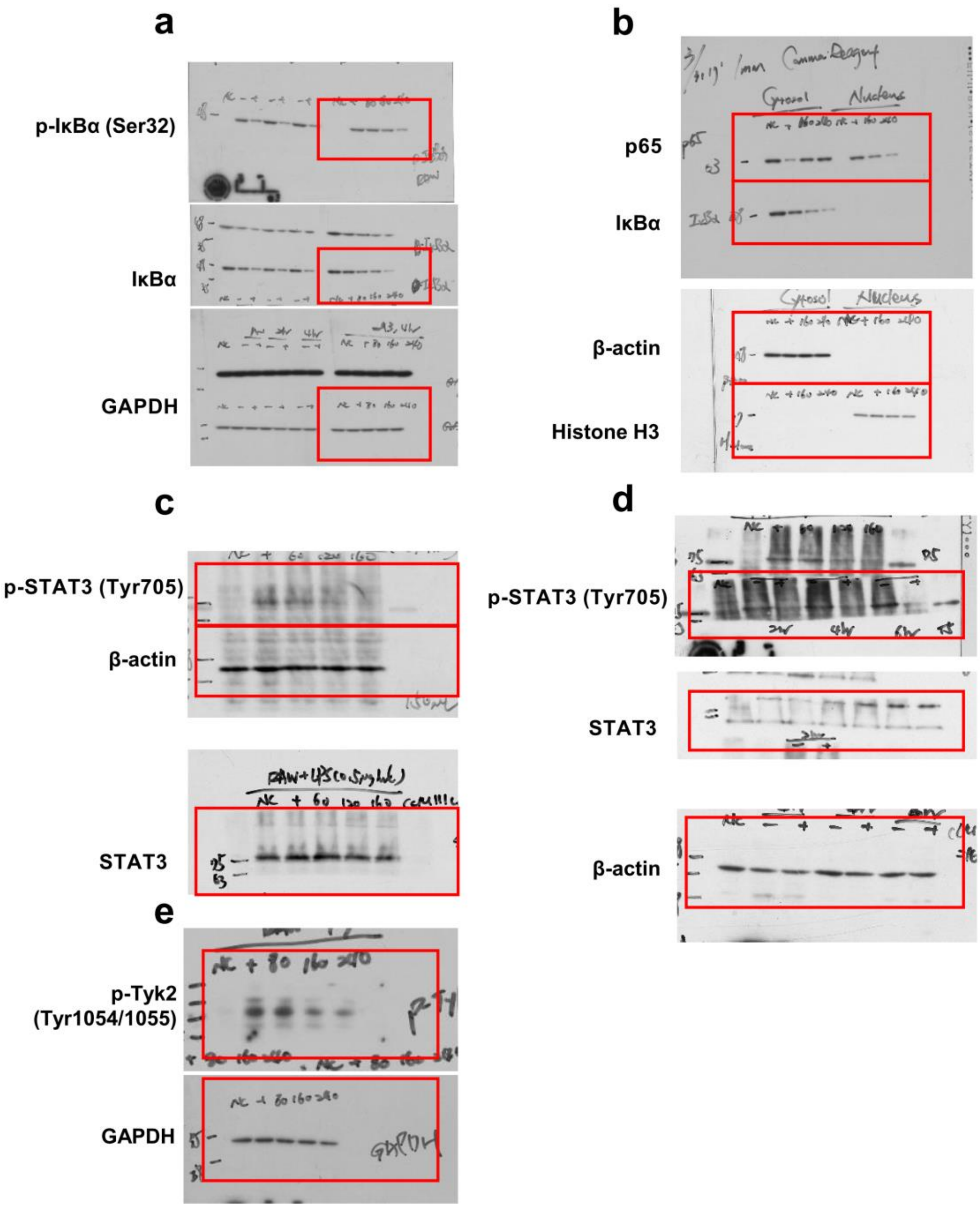

Supplementary Fig. 3

Full-length Western blot images in Fig.5.

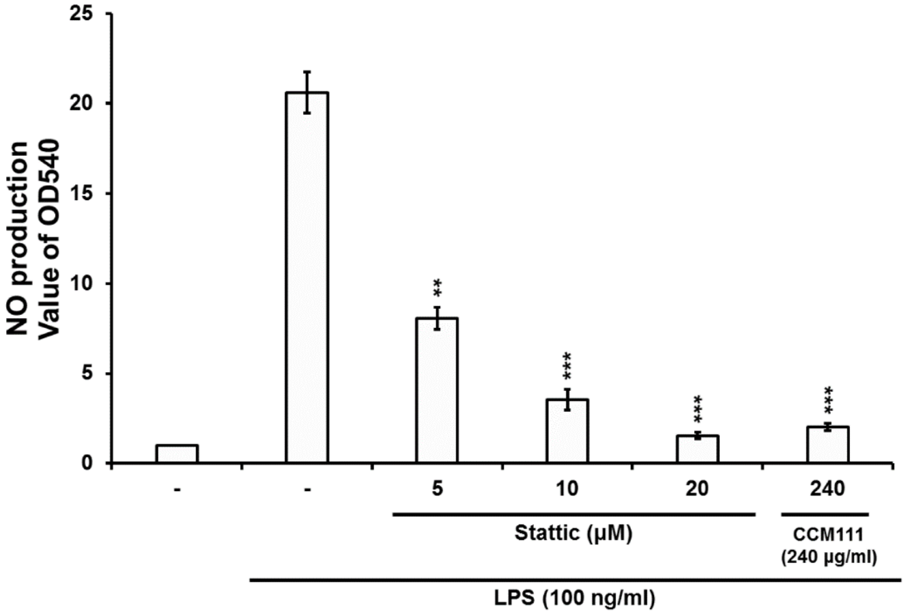

Supplementary Fig. 4

The effects of sattic and CCM111 on NO production and the protein expression of iNOS, COX-2, and inflammatory cytokines in murine RAW264.7 macrophages. The cells were treated with LPS (100 ng/ml) alone or in combination with different concentrations of stattic (0, 5, 10, 20 μM) and CCM111 (240 μg/ml). After incubation for 24 hours, the culture media was collected for nitrate assay analysis. The S.D. was performed by Student's *t*-test compared to the control or LPS only group. ***p*-value < 0.01 and *** *p*-value < 0.001. Results were obtained from three independent replicates.

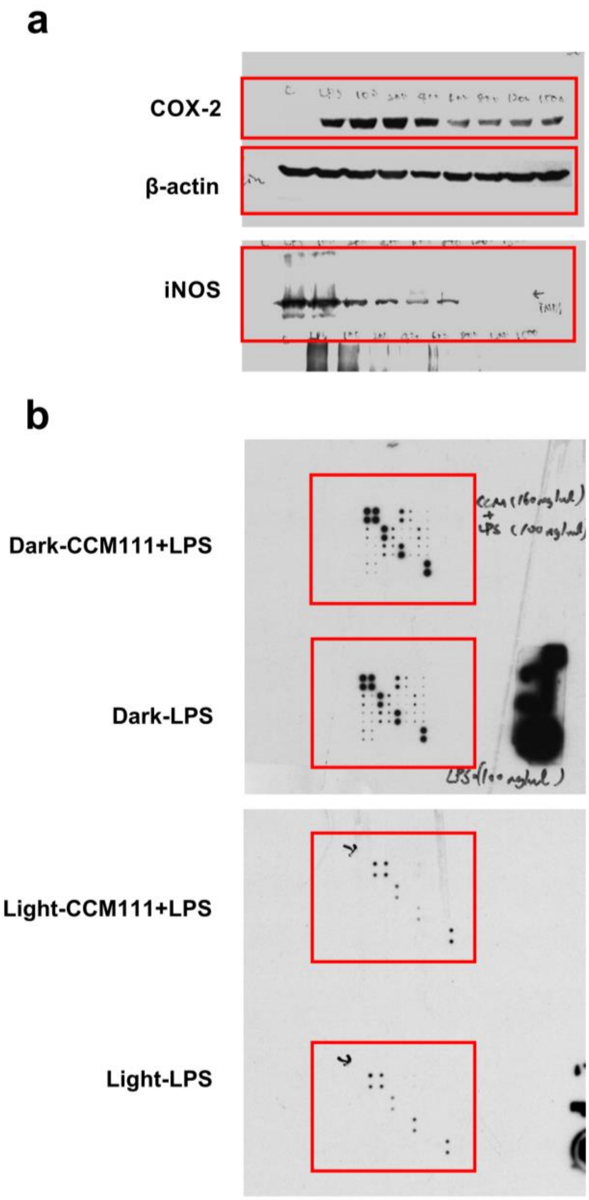

Supplementary Fig. 5

Full-length Western blot images in Fig.6.

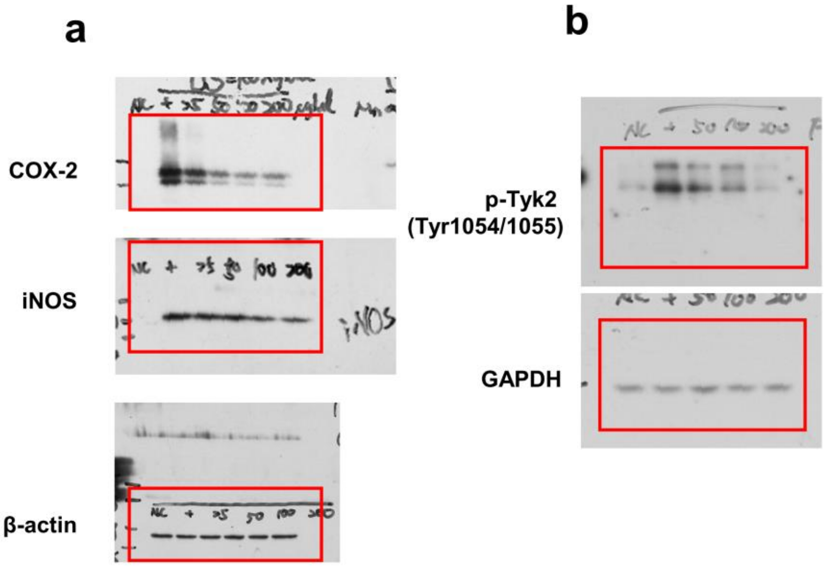

Supplementary Fig. 6

Full-length Western blot images in Fig.7.

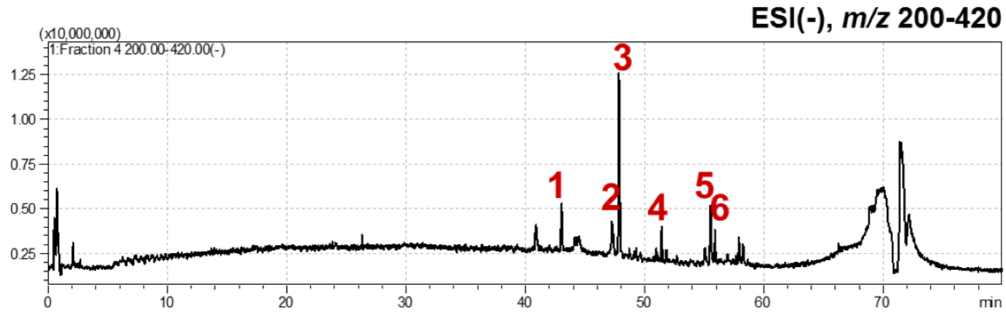

Supplementary Fig. 7

Qualitative analysis of Fraction 4. The Fraction 4 analyzed by HPLC-ESI-MS/MS. MS spectrum in negative ion mode of Fraction 4 was obtained. Six bioactive marker substances from ingredients of the Fraction 4 was qualitatively determined within 80 minutes under the selected LC/MS condition. antroquinonol B (peak 1), 14-deoxy-11,12-didehydroandrographolide (peak 2), antrocinnamomin C, (peak 3), 14-deoxyandrographolide (peak 4), methyl oleate (peak 5), and antrodin A (peak 6).

Supplementary Methods

Liquid chromatography-mass spectrometry (LC/MS) analysis. Fraction 4 was qualitatively determined within 80 minutes under selected LC/MS conditions. The LC/MS analytical system was followed previous study [1].

Reference

1. Lien, C. Y. *et al.* Oral treatment with the herbal formula B307 alleviates cardiac toxicity in doxorubicin-treated mice via suppressing oxidative stress, inflammation, and apoptosis. *OncoTargets and therapy* **8**, 1193-1210, doi:10.2147/ott.s82936 (2015).
